# Negotiating Multiple Minoritised Identities: An Interpretative Phenomenological Analysis of Intersecting Sexual Orientation, Gender Identity, and Suicidal Thoughts and Behaviours Among Transgender and Gender-Diverse Young Adults

**DOI:** 10.1007/s40653-026-00877-4

**Published:** 2026-04-21

**Authors:** Emma Rebecca Wallace, Siobhan O’Neill, Susan Lagdon

**Affiliations:** 1 Ulster University, Coleraine, United Kingdom; 2 Institute for Mental Health, University of Birmingham, Birmingham, United Kingdom

**Keywords:** Suicide, Trauma Exposure, Sexual Orientation, Gender Identity, Qualitative

## Abstract

Transgender and gender-diverse young adults, most of whom identify as lesbian, gay, bisexual, or queer, encounter considerable health, social, and systemic inequalities, leading to higher rates of trauma exposure, mental health difficulties, and suicidal thoughts and behaviours compared to their heterosexual and cisgender peers. The complex relationship between sexual orientation, gender identity, and suicide has received considerable attention, primarily from quantitative research focused on risk narratives, rather than an individual’s lived experiences or specific healthcare needs. The aim was to explore the lived experiences of transgender and gender-diverse young adults, highlighting the impact of navigating multiple minoritised identities, exposure to trauma, victimisation, unmet healthcare needs, suicidal distress, and barriers and facilitators to accessing mental health services and gender-affirming care. Six young adults from Northern Ireland (UK) were recruited via social media and participated in semi-structured interviews. Interviews were audio-recorded, transcribed verbatim, and analysed using Interpretative Phenomenological Analysis (IPA), following a process of coding, theme development, and interpretative synthesis. Five group experiential themes were identified: (1) Delicate peace, delicate progress; (2) Queer suffering, safety and otherness; (3) Queerness shaping ones’ worldview; (4) Unresolved trauma, and unmet healthcare needs; (5) Suicidal distress, and recovery. The findings elucidated two central narratives; the psychosocial impact of negotiating multiple minoritised identities in a heteronormative and cisnormative society and navigating suicidal distress by regaining a sense of control over your life. The findings provide valuable insights into the unique lived experiences, and specific healthcare needs of this population. Clinical implications are discussed, including the importance of trauma-informed care, cultural competence among healthcare practitioners, and tailored suicide prevention strategies for LGBTQ+ youth.

**Supplementary Information:**

The online version contains supplementary material available at https://doi.org/10.1007/s40653-026-00877-4.

## Introduction

Suicide is preventable and not inevitable. Nevertheless, suicide continues to pose a significant challenge to public health, representing the third leading cause of death among 15–29 year olds worldwide, with higher prevalence rates among marginalised groups (World Health Organisation, 2024). According to a national survey conducted in the United Kingdom, 73.1% of transgender and gender-diverse individuals also identify as lesbian, gay, bisexual, pansexual, or queer in terms of their sexual orientation (Government Equalities Office, 2018). Over the past twenty years, international research has highlighted significant mental health disparities, and elevated rates of suicidal thoughts and behaviours among LGBTQA+ young people (Gnan et al., 2019; McDermott et al., 2018; Rimes et al., 2019). Notably, it has been argued that suicide rates reflect broader systemic inequalities, as evidenced by the disproportionately high levels of suicidal distress observed among LGBTQA+ youth (Wallace et al., 2024). Indeed, international evidence indicates that LGBTQA+ young people are approximately three times more likely than their heterosexual or cisgender peers to have attempted suicide (di Giacomo et al., 2018; Hatchel et al., 2021; Williams et al., 2021). However, LGBTQ+ individuals are often treated as a homogeneous group, despite considerable diversity in their lived experiences and healthcare needs. In practice, marked health, social, and structural inequalities within this population are reflected in differing suicide incidence rates. For example, di Giacomo (2018) found that lesbian and gay young people were nearly four times more likely to have attempted suicide, while transgender youth were almost six times more likely than their heterosexual and cisgender peers. These disparities highlight the need for intersectional research to elucidate how interrelated social, psychological, and structural factors shape elevated suicide risk among LGBTQA+ young people.

International research indicates that 27% of all suicide attempts among LGBTQ+ young people occur before the age of twenty-one (Haas et al., 2011). Longitudinal studies suggest that suicidal thoughts and behaviours (STBs) commonly emerge during adolescence and may indicate an increased risk of suicide attempts; however, for some adolescents, suicidal behaviour can occur without preceding warning signs (Castellví et al., 2017). For LGBTQ+ youth, adolescence represents a critical period of vulnerability to STBs, characterised by significant biological, psychological, and social transitions, alongside the exploration of sexual orientation and gender identity (Hatchel et al., 2019; Marshal et al., 2011). The relationship between sexual orientation, gender identity, and STBs has thus generated much discussion, albeit primarily from quantitative studies concerned with discourses of risk, rather than an individual’s lived experiences or specific healthcare needs. Consequently, risk factors for suicide among LGBTQ+ young people are frequently categorised in a reductionist manner that fails to adequately capture the complexity, diversity, and significance of LGBTQ+ identities and lives (Cover, 2016; Nordmarken, 2023). Nevertheless, it is important to emphasise that suicide is a complex, multifactorial and highly contextual phenomenon, situated within historical, political and socio-cultural contexts (White & Morris, 2019). More broadly, international literature has drawn on the Minority Stress Theory (Meyer, 1995), to explain how external (distal) stressors like discrimination, victimisation, and rejection, along with internal (proximal) stressors such as internalised stigma or shame, contribute to poorer mental health outcomes and elevated suicide risk throughout the life span (Rogers et al., 2021).

A recent systematic scoping review identified a wide range of LGBTQ+ specific risk and protective factors for STBs among young people, spanning all levels of the socio-ecological model (Wallace et al., 2024). These included risk factors such as family and peer rejection, negative coming out experiences, bullying, interpersonal violence, and structural barriers to mental health and inclusive healthcare access. In addition, protective factors highlighted the importance of at-school and community safety, anti-bullying policies, family connectedness, and LGBTQ+ inclusive legislation and policies (Wallace et al., 2024). Building on this, McDermott et al. (2025) emphasised the need to understand how these factors manifest within the local context, noting that protective mechanisms such as affirming relationships, accessible support services, and identity-affirming environments are central to reducing suicide risk among LGBTQ+ youth. Notably, transgender and gender-diverse young people remain at substantially elevated risk even when sexual orientation is controlled for, underscoring the distinct impact of gender-identity related stressors (Kirakosian et al., 2023). Experiences of transphobia, gender-based victimisation, social exclusion, and restricted access to gender-affirming care are key psychosocial risk factors (Bird et al., 2024; Bochicchio et al., 2021). Awareness of these unique experiences underscores the importance of investigating risk and protective factors specific to transgender and gender-diverse populations in order to inform targeted, contextually appropriate, and evidence-based interventions.

In addition, a growing body of international evidence has highlighted the detrimental psychological health outcomes of traumatic stress, interpersonal trauma, childhood adversities, and post-traumatic stress disorder (PTSD) among LGBTQ+ young people (Ramos & Marr, 2023; Stenersen et al., 2019; Travers et al., 2020). For instance, increased prevalence rates of physical, sexual, and emotional abuse, as-well as neglect have been reported (Balsam et al., 2005; Tran et al., 2022), interpersonal trauma from discriminatory life events (House et al., 2011), and systemic oppression, all of which are associated with stress-related disorders and suicide risk (Nicholson et al., 2022; Smith et al., 2016). A significant nationwide survey in the United States regarding childhood adversities revealed that 83% of LGBTQ+ individuals had encountered one or more childhood adversities, while 52% had experienced three or more childhood adversities (Tran et al., 2022). In the United Kingdom, recent figures from Stonewall (2023) identified that hate crimes against transgender people have increased by 186%, and have typically involved physical violence or threats of violence. These concerning statistics coincide with a recent British Social Attitudes survey which highlighted that the United Kingdom is growing more discriminatory as a nation towards transgender and gender-diverse people (National Centre for Social Research, 2023). In 2020, NHS England commissioned an extensive independent review of gender identity services for children and young people (Cass, 2024). Despite facing considerable criticism regarding its methodology, scope, and recommendations (Horton, 2024; Noone et al., 2024), the review resulted in a nationwide prohibition on prescribing puberty blockers to transgender young people under eighteen (Department of Health, 2024). As a direct consequence, transgender and gender-diverse youth are still fighting to end the medicalisation of their bodies, and their lived experiences (Ward, 2021), with increasingly limited access to gender-affirming care.

Recent qualitative and mixed methods studies emphasise the importance of culturally congruent, contextually grounded understandings of suicide among transgender and gender-diverse young people (Marzetti et al., 2022; McDermott et al., 2018; Wright et al., 2021). This approach is particularly relevant in Northern Ireland, where the liberalisation of LGBTQ+ rights occurred much later than the rest of the United Kingdom, and where the implementation and social perception of these rights remain under-researched (Hayes & Nagle, 2016). Queerphobic prejudice, interpersonal violence, and discrimination have been argued to persist as common features of post-conflict societies, often replacing or migrating outright sectarianism (Duggan, 2017). Consequently, the peace process has been critiqued for failing to adequately improve LGBTQ+ lives, as it has not addressed the pervasive system of heteronormativity in Northern Ireland (Schubotz & O’Hara, 2011). Addressing suicide in this context therefore requires a social justice lens to understand its deep connections to structural and socio-cultural injustices (Button & Marsh, 2019). Centralising the narratives of those directly affected allows for recognition of the unique lived experiences of marginalised communities. Moreover, the multiple and intersecting forms of marginalisation associated with sexual orientation and gender identity underscore the need for intersectional research that can capture the complex systems of oppression shaping these young people’s experiences and suicide risk (Hereth et al., 2020; Sterzing et al., 2017). Such approaches engage with diverse and intersectional discourses that can help construct understandings of, and responses to, suicidal thoughts and behaviours that are culturally congruent and meaningful (White, 2017).

## Method

A qualitative Interpretative Phenomenological Analysis (IPA) was chosen for the overall study design, facilitated by in-depth semi-structured interviews which were flexible, trauma-informed, and open-ended. The study aimed to explore the diverse and unique ways in which LGBTQA+ young adults make sense of the complex intersections between sexual orientation, gender identity, and experiences of suicidal distress. IPA was selected due to its epistemological and methodological alignment with the study’s aims, providing a hermeneutic framework for understanding mean-making processes while remaining sensitive to the idiographic nuances of individual experience (Eatough & Smith, 2017; Larkin et al., 2011; Smith et al., 2022). A total of six participants were interviewed, consistent with recommendations for in-depth IPA studies (Smith et al., 2022). The principles of ‘sample size sufficiency’ were considered in relation to the richness and volume of data, qualities of the analysis, and pragmatic considerations (Vasileiou et al., 2018). Analysis proceeded on the understanding that findings are co-constructed through engagement between participant accounts and researcher interpretation. Rather than attempting full bracketing, we critically examined how our assumptions, and own experiences, shaped our analytic attention.

The first author conducted all interviews and led on analysis, drawing on prior clinical and research experience with LGBTQ+ young people in Northern Ireland. While this facilitated rapport and sensitivity to minority stress, trauma, and service navigation, it also risked foregrounding familiar explanatory frameworks. A reflexive journal was maintained throughout, particularly where interpretations felt intuitively aligned with trauma-informed or minority stress models. These were revisited during theme development to ensure claims remained grounded in participants’ language. Early coding clustered distress narratives primarily within trauma-related constructs. Through participant debriefing, we reconsidered whether this emphasis risked pathologising accounts and obscuring narratives of agency, belonging, and growth. Themes were subsequently restructured to better capture the co-existence of vulnerability and resilience within developmental contexts. Reflexivity was also enacted during interviews. Participants were invited to clarify or challenge interpretations in real time; in fact, many resisted assumptions of linear identity development, prompting greater analytic sensitivity to fluid and non-linear trajectories.

A decision log and reflexive journal was maintained to document analytic decisions and support methodological transparency. Throughout, we moved iteratively between idiographic case analysis and cross-case patterning, returning to full transcripts to test emerging claims against the integrity of individual accounts. Our interpretations are situated within contemporary and critical scholarship on sexual orientation, gender identity, and suicide; alternative readings are possible. The analysis aimed to provide a reflexive, contextually grounded account of how these young adults understood their identity and suicidal distress in Northern Ireland, attending to the interplay between personal meaning-making, developmental pathways, and the wider socio-political climate. Rather than isolating suicidality as individual pathology, we sought to situate participants’ accounts within relational, institutional, and cultural contexts, while preserving the idiographic integrity and complexity of each narrative discussed here.

### Participants

A purposive sampling strategy was used to recruit six LGBTQA+ young adults with lived experiences of self-reported mental health difficulties and suicidal thoughts and/or behaviours (STBs). The inclusion criteria stated that participants must (a) identify as LGBTQA+; (b) be aged between 18 and 24 years old; (c) reside in Northern Ireland; (d) have experienced mental health difficulties and STBs but no longer be at risk. Defining ‘at risk’, the exclusion criteria stated that participants should not participate if they were experiencing a high level of stress, anxiety, emotional distress and/or suicidal ideation. The first six participants who met the above criteria participated in the study. No financial compensation was provided to participants. The sociodemographic characteristics of participants are presented below (Table 1).

**Table 1:** Sociodemographic characteristics of participants

Demographics	Participants					
	Jamie	Max	Riley	Harper	Jack	Ash
Age	19	20	24	24	22	22
Pronouns	They/them	He/him	They/them	She/her	He/him	They/them
Ethnicity	White	White	White	White	White	White
Education	A levels	Degree	Degree	Degree	A levels	Degree
Disability	No	Yes	Yes	No	No	Yes
Gender identity	Fluid	Male (FTM)	Non-binary	Female (MTF)	Male (FTM)	Non-binary/transmasc
Sexual orientation	Queer	Bisexual	Bisexual	Straight	Bisexual	Lesbian
Suicidal ideation	Yes	Yes	Yes	Yes	Yes	Yes
Suicide attempt	No	Yes	Yes	Yes	No	No
Age of onset for suicidal ideation	11	Unsure	10	12	Unsure	Unsure
Age of first suicide attempt	-	15	17	14	-	-
Number of suicide attempts	-	4	1	4	-	-

### Data Collection

The recruitment strategy employed digital and print materials that were shared on social media and in the offices of partnered LGBTQA + and local mental health organisations. This strategy ensured a broad target population, and that young people were self-selecting to participate. The lead researcher developed a semi-structured interview schedule in collaboration with local LGBTQA+ organisations. The interview schedule focused on five key areas; (1) identity development, adolescence, and coming out; (2) intersectional identities and related experiences; (3) mental health difficulties and suicidal distress; (4) accessing mental health services and gender-affirming care; (5) Overcoming suicidal distress and opportunities for suicide prevention. For example, participants were asked about their background and identity, *“Can you describe what it is like for you to be an LGBTQ+ young person living in Northern Ireland?”*, and *“What does your sexual orientation and/or gender identity mean to you as a person?”.* They were also asked about their experiences of adversity and mental health challenges, for example, *“Have you ever experienced bullying or discrimination because you are LGBTQA+*,* and how did this impact your mental health?”.* Regarding suicidal thoughts and behaviours, participants were asked, *“If you feel comfortable*,* could you share your experiences with suicidal thoughts or behaviours and how these have affected your life?”.* Finally, participants were also asked about their coping strategies, resilience, and help-seeking. For example, *“What strategies or supports helped you manage these difficult experiences at the time?”.*

All prospective participants spoke with the lead researcher via telephone to confirm their eligibility. Following this, participants were e-mailed the participant information sheet, consent form and demographic monitoring forms which were returned via e-mail. All interviews were held online via video-conferencing software, and each interview lasted approximately sixty minutes. Before beginning the interview, all participants were asked to confirm that they were not currently experiencing a high level of stress, anxiety, emotional distress and/or suicidal ideation. All participants were offered follow-up care following their interview; although all participants declined this additional support. A signposting to services document was also e-mailed to all participants, and the vast majority of participants opted to receive an anonymised version of their transcripts following their interviews, with no changes requested.

### Data Analysis

The interview transcripts were analysed by the lead researcher using Interpretative Phenomenological Analysis (IPA) (Smith et al., 2022). Initial notation began with comprehensive line-by-line analysis with three discrete processes, including descriptive, linguistic and conceptual comments. This process ensured that salient phrases and developing experiential themes were derived directly from the dialogue, rather than pre-existing theoretical understandings. Reflecting a progressive interpretative process, initial exploratory notes were drawn together through meaning-making and clustered into experiential statements. In constructing personal experiential themes, salient features of statements were captured whilst reducing the volume of data. This process was repeated for each participant, with experiential statements bracketed between cases. Group experiential themes were developed by examining connections and shared meanings across cases, including higher order qualities. An experienced second researcher independently analysed a third of all transcripts, before the findings were shared in a data analysis session with all three authors. Any significant differences in interpretations or themes were fully debated before a consensus was reached on the final group experiential themes for this study.

### Ethical Considerations

Ethical approval was obtained from [UNIVERSITY NAME], School of Psychology Ethics Filter Committee and the University Research Ethics Committee (REC/23/0054). The study was conducted in accordance with the British Psychological Society’s (2018) code of ethics and conduct for undertaking research with human participants. Ethical procedures were further informed by contemporary literature on qualitative research with minoritised populations, emphasising the importance of balancing participant autonomy, safety, wellbeing, and the potential benefits of participation (James & Platzer, 1999; Mollard et al., 2020). The study was designed in accordance with trauma-informed guidelines for conducting qualitative research among vulnerable and/or marginalised groups (Alessi & Kahn, 2022). For example, it was crucial to strengthen participants’ capacity for choice, control and setting of personal boundaries. During the interviews, every effort was taken to avoid re-traumatisation by paying close attention to verbal and non-verbal cues that might indicate that a participant is reliving an experience, rather than recounting it. To mitigate any potential emotional distress, strengths-based questions were posed following any sensitive topics related to mental health, wellbeing, and suicidal distress.

Research with young people who have previously experienced suicidal distress requires careful consideration of safety and support mechanisms, including clear procedures for minimising distress and risk disclosures, which aligns with best-practice recommendations in the field (Barnard et al., 2021; Bhasin et al., 2022). For this reason, a structured ‘minimising harm and distress protocol’ detailed clear procedures for different scenarios; (a) participant is distressed; (b) participant discloses information that must be passed on; (c) participant discloses that they have an active suicide plan; (d) participant discloses that another person has an active suicide plan. Importantly, no disclosures or concerns regarding participants’ emotional wellbeing or safety arose during the interviews, and therefore the protocol did not require implementation. Given the ethical complexities inherent in suicide research with marginalised populations, additional measures were implemented to ensure confidentiality, use inclusive and identify-affirming language, and provide a safe environment for disclosure. After the interviews, participants were contacted via email to check on their wellbeing, offer additional support if needed, and provide information on relevant support services. This follow-up procedure was designed to prioritise participant safety, address any potential distress arising from the interview process, and ensure access to appropriate services. These procedures align with best practices reported in other qualitative studies exploring suicide among LGBTQA+ populations, which similarly emphasise the importance of providing follow-up care (Marzetti et al., 2022; McDermott et al., 2018). Notably, participants reported that no further support or follow-up care was required.

## Results

Five group experiential themes, each with four sub-themes, were drawn from the analysis and are presented in Table 2. The group experiential themes were endorsed by all participants, with varying degrees of convergence among sub-themes (supplemental). Pseudonyms are used to protect the anonymity of all participants.

**Table 2:** *Group experiential themes*,* and sub-themes*

Group experiential themes	Subthemes
1. Delicate peace, delicate progress	I. Delicate progress II. Religious teachings and norms III. A dangerous political climate IV. The importance of family acceptance
2. Queer suffering, safety and otherness	I. Adolescence and identity formation II. Queer safety in numbers; regretful camaraderie III. Inauthenticity, and stress of concealment IV. Expectations of a different life
3. Queerness shaping ones’ worldview	I. Shared trauma, and being othered II. Multi-faceted sense of self III. Acceptance, belonging and catalyst for change IV. Conflict and division within the queer community
4. Unresolved trauma, and unmet healthcare needs	I. Unresolved trauma cumulated in a breakdown II. Fighting to be believed III. Negative healthcare experiences IV. The importance of gender-affirming care
5. Suicidal distress, and recovery	I. Early onset of suicidal distress II. Lack of safety in ones’ suicidal distress III. Regaining a sense of control IV. Self-acceptance, and strong sense of self

### Delicate Progress, Delicate Acceptance

The first identified theme was *‘Delicate progress*,* delicate acceptance’*, which describes the slow progression of equality in Northern Ireland reflected within religious teachings and norms, the current political climate, and parental non-acceptance. From the outset, the contested nature of progress towards equality is alluded to by participants who question whether things have really changed, as illustrated:

*“Northern Ireland has its own political troubles and are probably still recovering from the aftershocks… There’s a kind of thing like we don’t have time to worry about queer issues because we can’t keep a government in… we have bigger fish to fry. We’re kind of shunted down the list*,* which is frustrating”.* (Jamie).

Harper also questions whether societal attitudes have meaningfully changed in Northern Ireland, or if people have become more adept at concealing their prejudice.

*“Things are better now that I am an adult… but I think that’s because I’m an adult rather than things changing all that much. Outwardly things have changed*,* but I don’t think society has changed that much. I think people just think they can’t outwardly say what they want to say now”.* (Harper).

For Jamie, there remains a *‘delicate’* or conditional form of acceptance for queer people, a blurring of difference contingent upon ones’ religious identity which is quite unique to Northern Ireland. For example:

*“It’s a very delicate form of acceptance. Once this person stops caring if I’m protestant or catholic*,* will my sexual orientation or gender identity be the next thing they go after or will they stay accepting”.* (Jamie).

Notably, the narratives of many participants suggested that Northern Ireland remains a society undergoing transition or transformation, marked by an absence of progression, acceptance, and space to be oneself engendered by exclusionary religious teachings, segregation, and a lack of inclusive education.

*“I grew up in an extremely politically conservative family*,* a protestant family. You know*,* I grew up believing that it was wrong to be gay because of the church… So being queer*,* it was very taboo”.* (Ash).

*“I had people who definitely held me at arm’s length at school or weren’t keen to be friendly because it was against their religion. Which I understand to a certain extent*,* but it doesn’t feel very Christian to not like people”.* (Riley).

In navigating religiosity, some disavowed religion entirely to protect the self; a memory that brought up feelings of anger, rejection and social isolation.

*“The main reason why I left religion was because I fully came to terms with the fact that I was*,* you know*,* queer. So*,* like if Christianity doesn’t want me to be queer*,* then I don’t want to be Christian”.* (Ash).

Returning to the slow progression of equality within Northern Ireland, participants challenged the *‘ambiguous’* nature of civic leadership, including the legality of conversion therapy. More broadly, participants also spoke about the *‘dangerous political climate’* which was later described as putting *‘trans lives at risk’*. For example:

*“I feel like the position of the government is quite ambiguous from my perspective. I don’t know if our government actually supports gay people… And*,* like*,* is conversion therapy still legal here?”*. (Riley).

*“Trans issues are very much an issue in Westminster*,* and it’s just human rights being stripped away from people. So*,* do we risk pushing for that here because what if we lose the progress that we’ve barely gotten”.* (Jamie).

Participants also reflected on the complex and difficult *‘journey towards acceptance’* that their parents experienced when they first disclosed their sexual orientation and/or gender identity. For some young people, there were negative and painful coming out experiences which negatively impacted their familial relationships. For example:

*“The first time I came out when I was twelve that affected me because I was being vulnerable and trying to talk to my mum*,* who just dismissed it. So*,* there was a lot of broken trust there”.* (Harper).

*“I was worried I’d get kicked out of the house. I had a bag packed*,* and it was a genuine fear at the time. My Mum is like mostly okay now*,* it’s kind of 50/50 on whether she will use my correct name or my dead name me”.* (Ash).

In contrast, the importance of family acceptance was demonstrated within participants’ positive coming out experiences, and through the rebuilding of these close relationships following earlier conflict or tension. Notably, this familial acceptance was even more important for those youth who felt a lack of belonginess, as illustrated:

*“Acceptance from my immediate family was amazing… It’s so important to have that support*,* especially in a world that is mainly against you”.* (Max).

*“I was going through a lot of stuff*,* they didn’t know how to help me… mental health and figuring myself out as a person*,* but we’ve come a long way and now we’re very close”.* (Jamie).

### Queer Suffering, Safety and Otherness

The second identified theme was *‘Queer suffering*,* safety and otherness’*, which demonstrates the stress of identity concealment and disclosure, along with traumatic experiences of bullying and a lack of social safety. In fact, every young adult had experienced pervasive bullying throughout their school and college years.

*“Being queer was hard because I was already bullied in school because I was kind of weird. I had undiagnosed autism when I was a kid. So*,* I was already being bullied for just existing*,* and then for being queer as-well”.* (Ash).

*“I’d say I was bullied especially in primary school I got called ‘gay’ a lot in a derogatory way… I never got into any fights*,* but as a teenager there were actually fights”.* (Harper).

Through Jack’s experiences as a trans man, we see the complex intersection of transphobia and misogyny, of *‘being infantilised’*, as illustrated:

*“In some places where I would have said ‘I’m trans’ there has been instant transphobia and misogyny*,* but also the sense of being infantilised. You know*,* being spoken over quite a lot*,* not allowed in certain places”.* (Jack).

Continuing with Jack, there was also an embodied expression of past victimisation, demonstrating the psychosocial impact of *‘queer suffering’*, and how traumatic experiences can be internalised to the self, causing mental distress.

*“It’s the grand total of having those thoughts of hopelessness in your head*,* like ‘you’re not doing enough’ or ‘you’re not being hypervigilant enough’. A lot of the anxiety or depression has come just from the struggles the community faces as a whole”.* (Jack).

The next passage cumulatively represents many of the participants’ experiences in public spaces; a perceived and sometimes realised lack of physical safety which was depicted as *‘queer safety in numbers; regretful camaraderie’*.

*“There’s a camaraderie that women have for other women*,* like we will do whatever to keep each other safe. There’s a very similar thing for queer people*,* particularly on a night out where we can’t be perceived as giving a reason for people to be violent*,* either verbally or physically with you… but*,* we wish we didn’t need to have that sense of community”.* (Jamie).

As illustrated in Harper’s account, experiences of intrusive or invasive questioning appear to destabilise a fragile sense of self, heightening the need for safety and vigilance. This heightened sensitivity leads to the internalisation of perceived judgement, producing deep feelings of shame. What emerges is a process in which shame functions not merely as an emotional response but as a protective strategy, guiding how participants negotiate social interaction and regulate their identities. Harper’s narratives demonstrates how these early negative experiences are turned inward as a form of hypervigilance or self-surveillance, constraining the possibilities for open or authentic expression and contributing to a persistent sense of otherness.

*“A lot of the questions they asked were invasive and sexual and that really negatively affected me. It filled me with quite a lot of shame. Like*,* make sure nobody knows about this*,* and I kept that*,* didn’t deal with it until I was in my twenties”.* (Harper).

### Queerness Shaping Ones’ Worldview

The third identified theme was *‘Queerness shaping ones’ worldview’*, which represents the shared experience of queerness shaping a multi-faceted sense of self. Participants experiences during adolescence exemplified this non-linear process of becoming.

*“I did the whole it feels like the gateway coming out. First off*,* I was a lesbian*,* then wait I’m trans*,* then the whole binary trans thing of ‘oh I must be straight’ you know as a trans man. But actually no*,* I’m bisexual*,* so it was really interesting now looking back at it”.* (Jack).

For Harper, the onset of puberty was a catalyst for exploring gender identity and expression. However, it also signalled the beginning of mental health difficulties and suicidal distress. Crucially, the next passage encapsulates an embodied sense of trauma, a feeling of being betrayed by ones’ body, and a sense of helplessness.

*“I would say I didn’t know I was trans when I was twelve*,* but puberty was starting to affect my body*,* and it was like ‘ew I don’t like this*,* somebody please help’*,* but nobody wanted to help”.* (Harper).

Among all participants, there was clear progression towards a multi-faceted and fluid sense of self and identity integration, with sexual orientation and gender identity becoming *‘just another part of me’.* For example:

*“Now it’s just kind of this thing about me*,* the same way I know what age I am*,* that I am queer… Now I am so much more sort of lax with it*,* because I realised that I don’t owe that part of myself to a person”.* (Jamie).

Nevertheless, the complex intersection of sexual orientation and gender identity remained evident within social contexts, reflected in experiences of gender performativity. This generated a heightened awareness of scrutiny and need to navigate self-presentation carefully. Jack’s account further illustrates how the forms of safety available to trans youth are often partial or conditional, sometimes compromised by hostility or exclusion, even with peer networks.

*“It was like my sexuality was making my gender identity invalid. I felt like I had been putting on a hyper masculine presentation for people*,* just so they would take me seriously”.* (Jack).

Participants spoke eloquently about the transformational role of the queer community in their lives; empowering and shaping the self. Queer spaces were described as *‘freedom from judgement and shame’*, nurturing a sense of belonging and acceptance:

*“I don’t know who I would be if I wasn’t part of the queer community because it’s such a fundamental part of what shaped me growing up. It shapes how you view the world for sure”.* (Riley).

However, the impact of conflict or division within the LGBTQA+ community in relation to trans inclusivity was also evident, both in terms of political rhetoric and its impact. Riley’s reflection extends Jack’s account of partial or conditional safety, illustrating the complex dynamics of community engagement; while profoundly supportive, they can simultaneously present challenges and barriers to belonging within the community.

*“And let’s not talk about the transphobia because it’s one of the worst… I feel like I’ve heard transphobia not just from straight people*,* but also gay people who feel like they shouldn’t be in the same community”.* (Riley).

### Unresolved Trauma, and Unmet Healthcare Needs

The fourth identified theme was *‘Unresolved trauma*,* and unmet healthcare needs’*, which illuminates the impact of unresolved trauma on participants’ mental health, leading to suicidal distress. Additionally, unmet healthcare needs are reflected within *‘fighting to be believed’*, characterised by anticipated and experienced stigma across different healthcare contexts. As shown, unresolved trauma had a significant impact:

*“I just bottled it up… I repressed it until I was at university*,* and by that summer I had a complete mental breakdown. My life just fell apart pretty much because I was having to deal with things”.* (Harper 5,24).

*“I definitely over-think so many things*,* my brain is constantly going two miles a second for no reason. Um. I guess I would say eccentric*,* but I don’t know if that’s just because of all the trauma I have*,* or if it’s who I am”.* (Max).

From Jamie’s account, there was the distressing struggle of *‘fighting to be believed’* across different help-seeking contexts, with an embodied *‘cry for help’* through disordered eating and chronic self-harm.

*“I ate less because I was so angry at my body for not getting thin enough to be a problem… I started self-harming more and more obviously. I would not wait to pull my sleeve down*,* hoping that my teacher would see and ask what was going on… The way I coped was I got sicker as a cry for help”.* (Jamie).

Despite numerous help-seeking experiences throughout adolescence, many young adults described *‘a vicious circle’* where their healthcare needs went unmet, contributing to their suicidal distress. Crucially, there were also missed opportunities for suicide prevention engendered by the contested, and stigmatised nature of trans healthcare within hospital settings. For example:

*“I’ve been through a lot of different counselling services*,* around that time I was initially with the school one but obviously at a catholic school*,* it was a religious counsellor so not particularly sympathetic in that sense… I did the whole rotating cast of counselling that didn’t really help”.* (Riley).

*“Zero support there… he was ‘ah I don’t think you have any problems*,* you’re just trans’*,* so he discharged me”.* (Harper).

In Jack’s case, these negative healthcare experiences continued within gender clinics where there was no access to gender-affirming care; the *‘root-cause’* of his mental health difficulties, and suicidal thoughts. Moreover, healthcare professionals were characterised as ‘ill-equipped’, possessing insufficient knowledge regarding trans identities and lives, hormone treatments, and referral procedures:

*“It has been just constantly seeing systems failing people*,* like I’ve accessed both gender clinics and not had any support or care from them. I had to go private*,* and I have to self-medicate hormones*,* and pay for my own blood tests”.* (Jack 6).

When questioned about how their lives might have differed if they had access to gender-affirming care and appropriate mental health support, Harper remarked:

*“My life would be so unimaginably different. There was a period of seven years that were absolute hell*,* and I wouldn’t have had to through that if any support had existed at all”.* (Harper).

### Suicidal Distress, and Recovery

The fifth identified theme was *‘Suicidal distress*,* and recovery’*, which explores the early onset of suicidal distress, and perceived lack of safety during ones’ suicidal crisis. Almost all participants also spoke of navigating suicidal distress and regaining a sense of control over their lives in unique and diverse ways. Furthermore, the youth in this study highlighted the significance of family and peer support, inclusive education, and mental health services tailored to meet the unique needs of LGBTQA+ young people.

*“In my childhood I remember having a lot of intrusive thoughts about death and suicide. When I was eleven or*,* even younger than that actually… it still sometimes pops back in*,* but I can tell when my mental health is starting to go down the drain because it comes to the forefront in my head more often”.* (Jamie).

From Jack’s account, we understand the importance of making sense of ones’ suicidal distress. For instance, there was an introspective awareness or understanding about the cyclical nature of depression, anxiety and suicidal thoughts.

*“I’ve been for a good part of my life been incredibly depressed*,* and anxious*,* and I’ve had cycles of that. I’ve always had suicidal thoughts and things like that*,* but never planned*,* never active situations”.* (Jack).

For Max, the pervasive impact of bullying victimisation was a primary cause of multiple suicide attempts during his school years.

*“It was already quite bad my mental health*,* and then in high school the bullying from younger years*,* I tried to kill myself. I think three times during high school*,* I didn’t end up in the emergency room*,* but there were close calls”.* (Max).

Significantly, many participants expressed that there was a lack of early intervention, and a perceived lack of safety in their suicidal distress, particularly once they had reached a crisis point. For example:

*“There’s such a large intersection of queer people with mental health issues*,* and they’re not getting the help they need…. Especially in rural areas there is that feeling of isolation*,* and when you’re isolated you might feel like there’s no-one there to stop you from hurting yourself*,* which is dangerous”.* (Jamie).

*“I had tried to… I had to go to the hospital for that*,* and the hospital was useless. They made sure I wasn’t dead*,* and then they made me promise I wouldn’t do it again*,* then right you can go home”.* (Harper).

The next passages speak to the individualised experience of navigating suicidal crisis, and *‘regaining a sense of control’*, including the conscious choice to stop attempting suicide, and the normalisation of ones’ responses to traumatic experiences.

*“When I was twenty-one*,* I tried again*,* but that time I was like*,* ‘I’ve tried this enough times*,* so I’m just going to give up’. It wasn’t until that*,* that I started to get better… it’s not improving my life*,* so I’m going to stop”.* (Harper).

*“I think one of the things that always helped was knowing that this is a normal reaction to a wild situation. Where we are isn’t normal and it shouldn’t be normalised*,* so of course I’m going to struggle”.* (Jack).

From Riley’s narrative, we see the importance of being able to access trauma-informed therapy which enabled them to process unresolved trauma, which significantly reduced the frequency and severity of suicidal thoughts:

*“To get to where I am now*,* I did 18 months of EMDR*,* that changed my life like I can’t even explain how good EMDR is. I have C-PTSD as-well*,* and oh my god I can’t express how good it was… Now*,* I don’t really have any suicidal ideation at all*,* no self-harming*,* nothing. It genuinely turned my life around”.* (Riley).

Importantly, the non-linear path towards recovery was evident throughout participants’ narratives and across all identified themes; understanding and processing unresolved trauma, peer support and familial acceptance, accessing gender-affirming care, and healing through self-compassion. As illustrated:

*“One of the biggest things that saved my life was just literally me transitioning which like is for most trans people”.* (Max).

*“I’m at a point of being extremely comfortable with being who I am*,* extremely comfortable with my queerness and like if that’s an issue for someone*,* that’s their problem. I’m not going to be afraid to make them comfortable”.* (Ash).

As discussed, meaningful support and acceptance from family and friends, along with finding *‘the right’* therapist or therapeutic intervention was crucial for most participants’ recovery:

*“Friends and family*,* and I do have a good therapist now too*,* she’s amazing but only because she’s also my generation. She’s the first therapist that’s really good… but there needs to be more support in terms of mental health*,* it’s just lacking everywhere”.* (Max).

In relation to unmet mental health needs, Jack also reflected on the significance of learning from young peoples’ challenging lived experiences. Additionally, participants stressed the importance of having services that were ‘*fit for purpose’*, able to meet the specific needs of LGBTQA+ young people, which was inextricably connected to having a sense of ownership over ones’ recovery. For example:

*“Listening to young people*,* actively listening and taking their frustrations*,* their problems and experiences on board. Also*,* build better systems that are fit for purpose for these young people*,* and make them feel like they are a part of something that they can actually change themselves”.* (Jack).

*“Put funding into these services so that practitioners are trained to deal with these things like gender issues*,* and the intersectionality of queerness and suicidality. Make it so we don’t have to explain our entire complex of our identities to someone because they’ve not been trained on how to discuss these issues”.* (Ash).

## Discussion

Among queer theorists it has been argued that identity is not in itself an attribute of selfhood, but rather a process of ‘becoming’, constructed in language, discourse, cultural norms, and practices of regulation (Cover, 2016; Butler, 1999). Participants’ narratives emphasise the inherently relational processes of identity development, especially within social contexts that amplified early experiences of difference from their peers. Encounters with ‘otherness’, manifested as social isolation, peer rejection, and a diminished sense of belonging, emerged as pivotal in shaping both identity and psychological or suicidal distress. Community affiliation played a key role in participants’ developmental journeys. LGBTQA+ spaces offered opportunities for identity exploration, affirmation, and mutual support, fostering resilience and thus reducing the risk of suicidal thoughts and behaviours (Beasley et al., 2015; Barr et al., 2016). However, participants’ accounts also demonstrate that LGBTQA+ communities are not uniformly protective; experiences of exclusion, transphobia, and erasure were sometimes present, particularly for those navigating multiple minoritised identities. This complexity highlights that social support can be both protective and conditional, underscoring the importance of examining relational dynamics and community contexts in understanding identity development. The findings underscore the enduring impact of ‘otherness’ on identity and wellbeing, highlighting the need for multi-level strategies, including inclusive education, culturally competent teaching, intersectional interventions, and structural reforms in mental health policy (Bhugra et al., 2023).

For the transgender youth in this study, the onset of puberty was a particularly challenging time, their bodies changing in ways that did not match their gender identity. Experiences of embodied trauma, including heightened anxiety and uncertainty about the future, align with evidence that delayed access to puberty blockers and gender-affirming care can exacerbate mental health difficulties (Horton, 2024a; Sorbara et al., 2020). Moreover, the findings demonstrate the psychosocial impact of negotiating multiple minoritised identities in a heteronormative and cisnormative society. Progress and acceptance were fragile, constrained by political conservatism, transphobic rhetoric, religious norms, and inadequate inclusive education, similar to research on queerphobia in post-conflict Northern Ireland (Duggan, 2013; Hayes & Nagle, 2016). However, this sense of thwarted belongingness also manifested in participants’ lives through an absence of family and peer support, familial conflict, and isolation, in accordance with existing literature (Askew, 2022; Chu et al., 2019; English et al., 2022). Indeed, the dynamic interplay between religiosity, cultural norms, exclusionary practices, and systemic marginalisation shapes mental health outcomes for LGBTQ+ youth, often compounding risk factors for psychological distress and suicidal ideation.

Significantly, participants also shared experiences of unique minority stressors for transgender and gender-diverse young adults. For example, the intersection of transphobia and misogyny, experienced as infantilisation and the pressure to adopt hyper-masculine behaviours to be taken seriously in public contexts. Similarly, non-binary and transmasculine participants reported repeated misgendering and deadnaming, contributing to identity invalidation. These experiences were accompanied by internalised stigma, shame, and queerphobia, reflected in anticipatory rejection, rumination, and stress of concealing one’s identity (Matsuno et al., 2022). Among transgender youth, the intersection of queerness, trauma exposure and suicidal distress was particularly pronounced. Suicidal distress often reflected collective struggles, perceived risk of victimisation, and chronic threat-vigilance or hypervigilance, a psychological mechanism linking stigma to internalising psychopathology (Ellis et al., 2016; Hollinsaid et al., 2023). The findings indicate that persistent internalised stigma and shame can contribute to gender identity repression, which may play a significant role in repeated suicide attempts during adolescence. Research on gender identity supression and the psychological pathways to suicide among transgender and gender-diverse young adults remains limited. However, identity-related shame and stigma also manifest through perceived burdensomeness, a key factor contributing to the development of suicidal thoughts and behaviours (Joiner, 2007; O’Connor & Kirtley, 2018).

The findings demonstrate that young people continue to face significant challenges in accessing timely, culturally sensitive and effective mental health services, despite the evidence that early, trauma-informed services can reduce suicide risk (Levenson et al., 2023). Participants described repeated attempts to seek help across primary care, statutory and non-statutory services, and emergency care, often experiencing delegitimisation, anticipated stigma, and a lack of cultural competence, which created a vicious circle of unmet needs. For many, prolonged exposure to these negative healthcare experiences exacerbated distress and unresolved trauma, with support only accessible through private counselling. Transgender participants reported similar difficulties in accessing gender-affirming care, including navigating specialist clinics, long waiting lists, and poorly coordinated pathways from primary care. Healthcare professionals were frequently described as ill-equipped, lacking knowledge of trans identities, hormone treatments, and referral processes, while normative about assumptions about gender and sexuality further delegitimised their healthcare needs. As a result, many young people were forced to seek private gender-affirming care, self-administer hormones, or rely on practices such as chest binding to manage embodied distress, often after years of untreated mental health difficulties and suicidal distress. These findings reinforce the urgent need to increase service accessibility, improve cultural competence among practitioners, and ensure timely, evidence-based pathways for both mental health and gender-affirming care to reduce suicide risk among LGBTQ+ youth (Higgins et al., 2025; Tordoff et al., 2022; Wright et al., 2021).

Most participants described early memories of intrusive suicidal thoughts, at such an early age whereby most could not comprehend what they were experiencing or why they were experiencing it. Participants also recalled suicidal distress which worsened gradually over time, situated within challenging heteronormative and cisnormative social contexts, a direct response to the increasingly unbearable psychological pain of being ‘othered’. Participants’ articulated that their suicidal distress was a response - a response to stigma, victimisation, and bullying, aligning with theoretical work on existential rejection and queer entrapment (Marzetti et al., 2022; McDermott & Roen, 2016). Several young adults explicitly described suicide as a form of escape, supporting the notion that suicidal queer youth, *“does not seek out death per se but seeks to escape from the complex tensions that are produced in subjectivation”* (Cover, 2016, p.9). The complex interaction of individual differences, socio-cultural factors and traumatic life events were most evident within the experiences of young adults who had survived a suicide attempt. Interpersonal risk factors, including loss, lack of school belonging, peer and parental non-acceptance, and bullying, were closely associated with increased suicide risk (Austin et al., 2022). The role of acquired capability, the ability to make a suicide attempt, was most apparent within these traumatic experiences, reflected within their heightened degree of fearlessness, impulsivity, and pain insensitivity (Joiner, 2007). Repeated suicide attempts appeared to involve a gradual desensitisation process, with increasing hopelessness, consistent with research on psychological habituation and dissociation as mechanisms for acquired capability (Shelef et al., 2017; Smith et al., 2015). These narratives highlight the significance of tailored crisis intervention services for LGBTQA+ young people; exemplified by the numerous missed opportunities for support and suicide prevention.

In addition, participants narratives highlighted the factors that shape and support the non-linear process of recovery, including navigating suicidal distress and regaining a sense of control. Recognising that suicidal behaviours were not improving their lives emerged as a significant turning point. Although intrusive suicidal thoughts often persisted as ‘warning signs’, this cognitive reappraisal represented a meaningful reclaiming of agency and the beginning of processing traumatic experiences. Indeed, a key analytic theme across the dataset was the role of trauma-informed care in supporting recovery and normalising responses to traumatic experiences, highlighting pathways to healing for LGBTQA+ youth. Trauma-informed approaches can address identity-specific marginalisation, minority stress, and other forms of trauma, ensuring support is validating and culturally sensitive (Levenson et al., 2023; Hall & DeLaney, 2021). Across the sample, recovery was closely linked to self-care strategies, coping methods, and a sense of purpose, including engagement with and support for other LGBTQA+ youth. These findings underscore the importance of trauma-informed and accessible gender-affirming care, particularly given legislative restrictions that hinder timely support and suicide prevention among transgender and gender-diverse youth (Price et al., 2023; Turnbull-Dugarte & McMillan, 2022). They also highlight the need for strengthened civic leadership to counter harmful political discourses, alongside inclusive, culturally competent healthcare practitioners equipped to address the specific healthcare needs of LGBTQA+ young people. Overall, promoting wellbeing in this population requires coordinated interventions across individual, community, and structural levels that attend to both identity-specific and systemic factors.

While this study provides novel insights into the intersection of sexual orientation, gender identity, and suicidal thoughts and/or behaviours among LGBTQA+ young people, several limitations should be considered when interpreting the overall results. First, commonly discussed constraints of Interpretative Phenomenological Analysis (IPA) include its intrinsic subjectivity, typically smaller sample sizes, and limited generalisability. However, the objective of IPA is not to generalise, but to provide comprehensive, nuanced, and meaningful insights into subjective lived experiences. The interpretative nature of IPA is further reflected in its double hermeneutic approach, in which both researcher and participant contribute to the interpretation of experiences. A purposive sampling method was employed to recruit LGBTQA+ young adults who had self-reported prior experiences of mental health difficulties and suicidal distress. This non-random sampling method entails selecting participants based on specific experiences, which may not reflect the broader LGBTQA+ population. Additionally, purposive sampling can introduce homogeneity depending on the criteria used; however, the strategy effectively captured participants who were lesbian, gay, bisexual, or queer, and transgender or gender-diverse. Finally, recruiting primarily through digital social media may have inadvertently excluded young people with limited online access, including those from lower socioeconomic backgrounds.

## Conclusion

This qualitative study represents the first empirical investigation concerned with the complex intersection of sexual orientation, gender identity, and STBs among transgender and gender-diverse young people in Northern Ireland, United Kingdom. The results represent a significant contribution to knowledge, enhancing our understanding of intersectional marginalised identities, the current socio-cultural and political climate, identity formation and development, the role of stigma and discrimination, unique minority stressors, and suicidal distress. Furthermore, the findings demonstrate the numerous barriers to accessing mental health, crisis intervention, and gender-affirming care in the United Kingdom, a significant yet under-researched area of research. Through its examination of diverse queer identities and suicide narratives, the findings provide important evidence and clinical implications that may inform tailored suicide prevention initiatives which are responsive to the specific healthcare needs of LGBTQA+ young people. In addition, the clinical implications reflect the significance of accessible and inclusive healthcare, trauma-informed care, cultural competency among mental health practitioners, early intervention, prevention and treatment, as-well as tailored crisis intervention services. Lastly, the findings demonstrate the considerable challenges that exist within our communities, educational institutions, and healthcare environments that must be addressed in order to reduce the existing health disparities among LGBTQA+ youth.

## Supplementary Information

Below is the link to the electronic supplementary material.

Supplementary Material 1

## Data Availability

The raw data, including transcripts of interviews, cannot be openly released due to ethical concerns regarding participant confidentiality. Processed data, such as thematic analyses, are available upon reasonable request, subject to ethical review and participant consent.
