# Supplementary material for: Negotiating Multiple Minoritised Identities: An Interpretative Phenomenological Analysis of Intersecting Sexual Orientation, Gender Identity, and Suicidal Thoughts and Behaviours Among Transgender and Gender-Diverse Young Adults
Source: J Child Adolesc Trauma. 2026 Apr 21;19(3):1831–46. doi: 10.1007/s40653-026-00877-4 (PMC13546265; doi:10.1007/s40653-026-00877-4)
Supplement: Supplementary file 1 [file 40653_2026_877_MOESM1_ESM.docx]

**Negotiating multiple minoritised identities: An Interpretative Phenomenological Analysis (IPA) exploring the complex intersection of sexual orientation, gender identity, and suicidality among transgender and gender-diverse young adults**

**Supplemental 1**

*Thematic mapping across all participants*

| **Group experiential themes, with subthemes** |  |  |  |  |  |  |
| --- | --- | --- | --- | --- | --- | --- |
|  | Jamie | Max | Riley | Harper | Jack | Ash |
| 1. Delicate progress, delicate acceptance |  |  |  |  |  |  |
| Delicate progress, delicate acceptance | Yes | Yes | Yes | Yes | Yes | Yes |
| Religious teachings, and norms | Yes | - | Yes | - | - | Yes |
| A dangerous political climate | Yes | Yes | Yes | Yes | Yes | Yes |
| Family acceptance in a world that is against you | Yes | Yes | Yes | Yes | Yes | Yes |
| 2. Queerness shaping ones’ worldview |  |  |  |  |  |  |
| Adolescence and identity formation | Yes | Yes | Yes | Yes | Yes | Yes |
| Multi-faceted sense of self | Yes | Yes | Yes | - | Yes | Yes |
| Acceptance, belonging and a catalyst for change | Yes | Yes | Yes | Yes | Yes | Yes |
| Conflict and division within the queer community | - | - | Yes | - | Yes | - |
| 3. Queer suffering, safety and otherness |  |  |  |  |  |  |
| Shared trauma and being othered | Yes | Yes | Yes | Yes | Yes | Yes |
| Queer safety in numbers; regretful camaraderie | Yes | Yes | Yes | - | - | Yes |
| Inauthenticity, and stress of concealment | Yes | Yes | - | Yes | Yes | Yes |
| Expectations of a different life | - | Yes | - | Yes | Yes | - |
| 4. Unresolved trauma, unmet healthcare needs |  |  |  |  |  |  |
| Unresolved trauma cumulated in a breakdown | - | Yes | Yes | Yes | - | - |
| Fighting to be believed | Yes | Yes | - | Yes | Yes | - |
| Negative healthcare experiences | Yes | Yes | - | Yes | Yes | - |
| Importance of gender affirming care | Yes | Yes | - | Yes | Yes | Yes |
| 5. Suicidal distress, transforming the self |  |  |  |  |  |  |
| Early onset of suicidal distress | Yes | Yes | Yes | Yes | - | - |
| Lack of safety in ones’ suicidal distress | Yes | Yes | - | Yes | - | - |
| Regaining a sense of control | Yes | Yes | Yes | - | Yes | - |
| Self-acceptance, and strong sense of self | Yes | Yes | Yes | - | Yes | Yes |
